# Antipsychotic Drugs Efficacy in Dextromethorphan-Induced Psychosis

**DOI:** 10.3390/biomedicines11010123

**Published:** 2023-01-03

**Authors:** Malgorzata Zaremba, Pawel Serafin, Patrycja Kleczkowska

**Affiliations:** 1 Military Institute of Hygiene and Epidemiology, 01-163 Warsaw, Poland; 2 Department of Experimental and Clinical Pharmacology, Centre for Preclinical Research (CBP), Medical University of Warsaw, 02-097 Warsaw, Poland; 3 Maria Sklodowska-Curie, Medical Academy in Warsaw, Solidarnosci 12 Str., 03-411 Warsaw, Poland

**Keywords:** dextromethorphan, toxicity, robotripping, antipsychotics, neuroleptics, psychosis

## Abstract

Psychosis is known as a broad term of symptoms that cause serious disorganization of behavior, thinking, and perception of reality. One of the medicines that recently gained much attention in terms of its psychotic potential is dextromethorphan (DXM). DXM, a widely used antitussive drug, is a commonly abused drug because of its euphoric, hallucinogenic, and dissociative properties. To date, DXM is a legally marketed cough suppressant that is neither a controlled substance nor a regulated chemical under the Controlled Substances Act. The management of DXM-related psychosis is dependent on the type of psychotic symptoms. Atypical neuroleptics (i.e., olanzapine, risperidone, quetiapine) and typical haloperidol have been used in symptomatic treatment due to their efficacy, especially in positive symptoms (hallucinations and delusions). These agents are also recognized as the preferred option in the symptomatic treatment of DXM-related psychosis due to their better efficacy and safety profile than typical haloperidol in the short-term course. The focus of the present review concerns the current stage of knowledge about DXM psychotic potency as well as the management of DXM-related psychoses with a special emphasis on atypical antipsychotic drugs (i.e., olanzapine, risperidone, quetiapine, and haloperidol).

## 1. Introduction

From the pharmacological point of view, dextromethorphan (DXM) is the methylated dextro-isomer of levorphanol, a codeine analog, and the most commonly used over the counter (OTC) dry cough suppressant. DXM is also commercially available in combination with other substances: acetaminophen, decongestants, antihistamines, as well as guaifenesin in numerous cough and cold medications. Recently, DXM with quinidine in a combo pill has also been indicated in the treatment of emotional lability—pseudobulbar affect accompanies amyotrophic lateral sclerosis (ALS), multiple sclerosis (MS), Alzheimer’s disease, and other dementias [1,2].

Indeed, DXM was first introduced into clinics in 1958 as a safe replacement for codeine. Between the 1960s and 1970s, the tablet forms of DXM began to be recreationally misused due to their euphoric, hallucinogenic, and dissociative properties. Nowadays, there are approximately 140 products that contain DXM available worldwide, including liquid-filled capsules, tablets, oral strips, and lozenges, given its clinically significant advantage over many other marketed cough suppressants. To fight addiction, drug companies have developed a liquid form of the drug (syrup), which requires larger quantities to obtain central nervous system (CNS) side effects; however, the problem of abuse has not been resolved.

The emergency departments in the U.S. have recorded approximately 6000 visits a year [3] due to DXM toxicity, and most importantly, more than half of such visits occur in adolescence [4]. According to the 2006 report of the Office of Applied Studies, Substance Abuse and Mental Health Services Administration (SAMHSA), approximately 3.1 million people in the U.S. aged 12–25 were intoxicated with OTC cough or cold medications containing DXM [5]. This may be due to the availability of the OTC drug as well as online information on how to “trip” successfully on DXM. Data from the AAPCC’s National Poison Data System showed that the annual rate of intentional abuse of DXM tripled from 2000 to 2006, then plateaued from 2006 to 2015 [6]. Similarly, according to the European Monitoring Centre for Drugs and Drug Addiction, the diversion and non-medical use of OTC medicines have increased during the last decade, probably due to OTC drugs being considered more socially acceptable, less stigmatizing, and “safer” than illicit substances. The problem mostly includes the recreational use of cough syrups containing codeine or DXM, and some decongestants (e.g., pseudoephedrine) [7].

There is currently no federal ban or restriction on DXM sales. Some individual states in the U.S. limit the quantity of DXM sold or restrict sales to those under the age of 18. To date, fourteen states in the U.S. have prohibited the sale of DXM-containing products to minors, and the FDA has considered increasing sales restrictions [8,9]. However, the FDA’s Drug Safety and Risk Management Committee voted against scheduling DXM in 2010 citing the drug’s economic and public health benefits in reducing cough symptoms [10].

Recreational use of high-dose DXM is known as robotripping (from Robo—DXM street name and Robitussin—a cough syrup brand name) [11]. Indeed, some of the effects observed by subjects who abused DXM are described as follows: “*I walk like … a robot. Identically. I feel relaxed, but I walk like some handicapped cyborg. My legs are a bit straightened, slightly bent at the knees, and walking is done on the principle of lifting one leg in this position all the time, it gives a few cm a step forward and so on alternately*” [12].

Notably, apart from the characteristic way of walking, DXM-containing remedies were found to exert a state of psychosis [13,14]. Among others, this side effect is quite common, transient, and poorly responds to antidotes. The appropriate drugs with the potential to reduce DXM-induced psychotic states are neuroleptics (also known as antipsychotics) mainly from the atypical group. Conventional/typical antipsychotics exert their effects primarily via the D2 receptor blockade, while atypical antipsychotics act mainly as serotonin–dopamine antagonists of both D2 and 5-HT2A receptors (please see Section 4) [15].

In addition, the problem of DXM misuse and psychosis is aggravated by the fact that numerous tips on the internet describe ways to extract DXM from other DXM-containing mixtures, and some of these were presented by Pascali et al. [16]. OTC products with DXM often contain other ingredients, such as acetaminophen, chlorpheniramine, and guaifenesin, that have their side effects, such as liver damage, rapid heart rate, lack of coordination, vomiting, seizures, and coma. To circumvent the many side effects associated with these other ingredients, a simple chemical extraction procedure has been developed and published by DXM abusers via the internet. In fact, a simple single- or two-stage acid–base extraction has been described using household products, such as ammonia, lighter fluid, and lemon juice, that results in a powder (i.e., Crystal Dex) or a liquid (i.e., DXmeon juice) form, respectively [17].

Therefore, in this review article, we outline the current stage of knowledge about DXM psychotic potency with a particular emphasis on the possible management and thus efficacy of antipsychotic drugs.

## 2. Pathophysiology of DXM Toxicity

Although structurally related to opioid agonists, DXM is a weaker agonist of mu-opioid receptors, mostly in overdose [18]. Thus, DXM does not appear to induce typical opioid-related side effects, such as dependence and respiratory depression, at therapeutically active concentrations.

Similar to ketamine and phencyclidine (PCP), DXM primarily acts through N-methyl-D-aspartate (NMDA) receptors, leading to a risk of psychotic symptoms, such as hallucinations, dissociation, euphoria, agitations, and even coma [3,19]. Interestingly, DXM exhibits higher affinity as an NMDA receptor antagonist than ketamine in some cellular assay systems [20].

**Figure 1:**
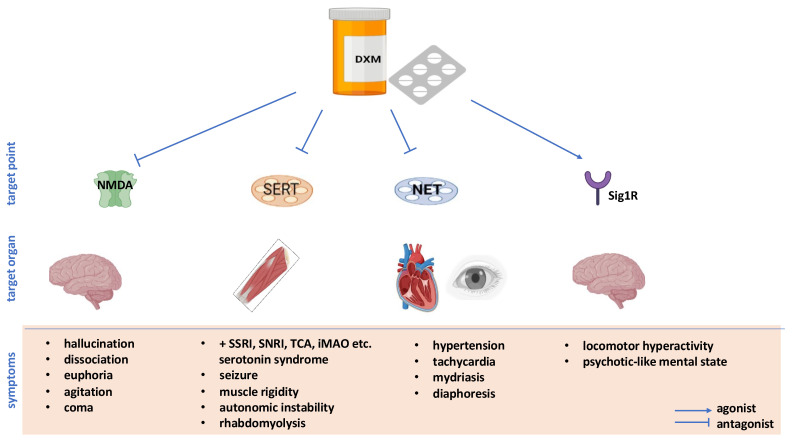
Dextromethorphan mechanisms of action and related side effects. Abbreviations: DXM—dextromethorphan; NMDA—N-methyl-d-aspartate (NMDA) receptors; SERT—serotonin transporters; NET—noradrenaline transporters; Sig1R—sigma-1 receptors; α3β4N—alpha3-beta-4 nicotinic (nACh) receptors; SSRI—selective serotonin reuptake inhibitors; SNRI—noradrenaline reuptake inhibitors; TCA—tricyclic antidepressants; iMAO—monoamine A inhibitors. Desired action and side effects are presented on an either white or red background, respectively. Created with BioRender.com.

In addition, the active metabolite of DXM (i.e., dextrorphan) exerts a variety of physiological effects via several mechanisms besides NMDA. Dextrorphan (DEXO) is a multi-target compound that tackles, among others, sigma-1 receptors (Sig1-R) and serotonin transporters (SERT). Furthermore, by inhibiting noradrenaline reuptake and blocking alpha3-beta-4 nicotinic (nACh) receptors, DEXO may increase norepinephrine availability in the synaptic cleft [20,21]. However, the mechanism by which DXM’s receptor agonism and antagonism translate to a clinical effect is not well understood [22]. The whole view of possible consequences that may occur in the settings of DXM’s overuse is shown in Figure 1.

Psychotic symptoms (i.e., hallucinations, euphoria, dissociation, agitations, and coma) are the main result of NMDA receptor blocking [3]. Furthermore, the SERT blockade and the increased serotonin pool in the synaptic clefts may lead to the potential risk of serotonin syndrome (seizures, muscle rigidity, autonomic instability, and rhabdomyolysis) [23,24,25]. Such activity is highly probable in eighter, the settings of overdose or standard doses when combining DXM with numerous antidepressive serotonergic agents (i.e., selective serotonin reuptake inhibitors—SSRIs, noradrenaline reuptake inhibitors—SNRIs, monoamine inhibitors—MAOIs, tricyclic antidepressants—TCAs) and opioids with selective serotonin reuptake inhibition potency (i.e., tramadol, methadone, fentanyl), as well as cocaine or other CYP2D6 inhibitors [24,25,26,27]. Also, the dietary supplements tryptophan and St. John’s wort are thought to be implicated in the appearance of such syndromes with DXM [28].

The metabolite DEXO also binds to noradrenaline transporters (NETs), leading to diminished reuptake of peripheral adrenergic neurotransmitters and causing hypertension, tachycardia, mydriasis, and diaphoresis (Figure 1) [3,29]. In addition, DXM binds sigma opioid receptors in the medulla, thus leading to desired antitussive effects [18].

## 3. Treatment of DXM Toxicity—Role of Antipsychotics

### 3.1. DXM Toxicity

Consider the axiom of Paracelsus “*All things are poison and nothing (is) without poison; only the dose makes that a thing is no poison*”; it is known that the dose makes the compound harmful [30]. Consequently, DXM, similar to other clinically used drugs, is not free from undesirable actions. Depending on the ingested dose, DXM toxicity can have a wide range of symptoms [3]. According to the published data, four specific levels (plateau) of DMX intoxication have been described (Table 1) [31]. However, numerous data have shown that DXM exerted infrequent and usually mild adverse reactions [32], whereas Logan and co-workers together with other researchers demonstrated serious long-term sequelae for DXM, such as assault, suicide, and homicide [33,34,35]. It is important to consider that the risk of psychosis may be the result of drug interactions, even greater when DXM is combined with other CNS depressants (e.g., benzodiazepine, non-benzodiazepine sedatives/hypnotics, anxiolytics, tranquilizers, muscle relaxants, general anesthetics, opioids, and alcohol-containing products).

### 3.2. Psychosis Induced by DXM Intoxication

DXM possesses the ability to induce visual hallucinations and paranoia. Price and Lebel demonstrated that DXM exerts delusions of telepathy and hallucinations, which are exposed as the ability to communicate with people without speaking or to “see into people”, respectively [13]. Similar effects have been shown by Miller in 2005, where a woman with a lack of personal or family psychiatric history of psychoses and intoxicated with DXM (2400–3600 mg per day) was characterized. She experienced both the delusional belief of having the ability to communicate with aliens and tactile hallucinations of “fiber” rolling around under her skin [37]. Similarly, in the next study, DXM was revealed to induce auditory hallucinations in a 66-year-old male that suggested he hurt or even kill himself [38]. Possible violent behavior was also reported by Modi et al. [34] in the case report of a woman who abused DXM and heard voices compelling her to kill herself and her aunt. Recently, Bernstein and colleagues published similar findings, where high doses of DXM were the causative factor that induced delusions and hallucinations, culminating in the patient’s violent and self-destructive acts.

Notably, concomitant use of DXM with other drugs in cough or cold remedies may result in a high risk of intoxication [39]. A good example was presented by Leighton and further by Sullivan et al., who demonstrated that pseudoephedrine potency causes paranoic psychotic states and auditory hallucinations at high doses [40,41].

However, most cases presented DXM toxicity at relatively high doses, whereas some papers have shown its hallucinogenic activity at a registered, antitussive dose (20–30 mg up to four times a day in divided doses) [42]. In immediate-release DXM formulations, standard doses range from 5 mg to 30 mg with a maximum amount of 120 mg/day [43]. In extended-release formulations, 60 mg is the standard dose with a maximum amount of 120 mg in 24 h. It should be also emphasized that patients are unable to give a clear history and the dose of DXM used due to altered mental status. Indeed, Nairn and Diaz [44] showed that DXM in the dose of 80 mg was able to induce moderate toxicity including hallucinations in an 8-year-old-boy; however, the potentially harmful effect has also been associated with the combined use of pseudoephedrine hydrochloride (500 mg). Notably, when DXM is administered at doses of 300 mg to 600 mg, dissociation and coma can occur [43].

In another case study, severe acute psychosis (visual and auditory hallucinations) diagnosed in young girls was associated with concomitant use of an over-the-counter formulation of pseudoephedrine with DXM and other compounds (e.g., steroids, carbinoxamine) [45].

**Figure 2:**
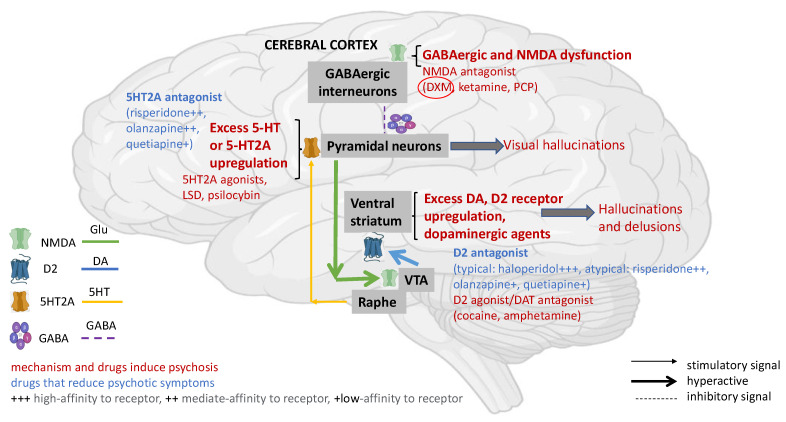
Potential drug-related psychosis pathways. Neurobiological and pharmacological evidence has revealed that psychosis symptoms, such as hallucination and delusions, are mediated by overactivation of the mesolimbic pathway, while visual hallucinations are consequences of visual cortex overstimulation. NMDA receptor dysfunction, excessive serotonin (5-HT) release, or 5-HT2 upregulation in the cerebral cortex may be the causes of further sustained activation of pyramidal neurons and diminished glutamatergic (Glu) input to the ventral tegmental area (VTA). Next, dopamine (D2) receptor upregulation and excessive dopamine (DA) release in the ventral striatum lead to hallucinations and delusions. Abused drugs and substances, including NMDA antagonists (i.e., dextromethorphan-DXM, ketamine, phencyclidine-PCP), psychedelic 5-HT-2A receptor agonists (i.e., lysergic acid diethylamide-LSD, psilocybin), psychostimulants—D2 agonist/DAT antagonists (i.e., amphetamine, cocaine), all have been reported to trigger hallucinations and delusions. Conversely, antipsychotics with both D2 and 5-HT2 high affinity have been shown to effectively treat drug-related psychotic symptoms. Adapted with modification from [46]. Created with BioRender.com.

The causative role of DXM in psychosis is still elusive. Neurobiological and pharmacological evidence has revealed that psychosis symptoms, such as hallucinations and delusions, are mediated by overactivation of the mesolimbic pathway, while visual hallucinations are consequences of visual cortex overstimulation [46]. Among others, NMDA receptor dysfunction, excessive serotonin enhancement, or 5-HT2 upregulation in the cerebral cortex may be the main causes underlying DXM psychotic potency (Figure 2) [47,48,49]. In addition, DXM is metabolized by the CYP2D6 system; thus, fast or ultra-metabolizers (nearly 85% of the US population) may be more susceptible to unintentional overdose and psychosis as well [50].

## 4. Antipsychotics—General Overview

The first antipsychotic drug was chlorpromazine, discovered in 1951 as an anesthetic adjunct, which was shown to be effective in reducing the positive symptoms of schizophrenia; however, multiple side effects were noted, including extrapyramidal syndromes (EPS) such as dystonia and tardive dyskinesia. Further research resulted in the discovery and introduction into the clinics of butyrophenones with the best-known agent in the group being haloperidol. This group was characterized by better effectiveness against the positive symptoms of schizophrenia with much fewer adverse reactions. Unfortunately, neither phenothiazines (i.e., chlorpromazine, trifluopromazine, thioridazine, trifluoperazine, etc.) nor butyrophenones (i.e., haloperidol, trifluperidol, droperidol) were effective in treating the negative and cognitive symptoms [51].

Due to the risk of unpleasant and sometimes lifelong EPS, newer antipsychotic medications than haloperidol have been discovered and formulated. The rapid dissociation of drugs from dopamine D2 receptors is a plausible explanation for the improved EPS profile of atypical antipsychotics. In 1990, the first atypical drug, clozapine, was developed. Several reports revealed that clozapine was superior to other marketed antipsychotics in terms of its efficacy, especially in the treatment of refractory schizophrenia [52,53,54].

**Figure 3:**
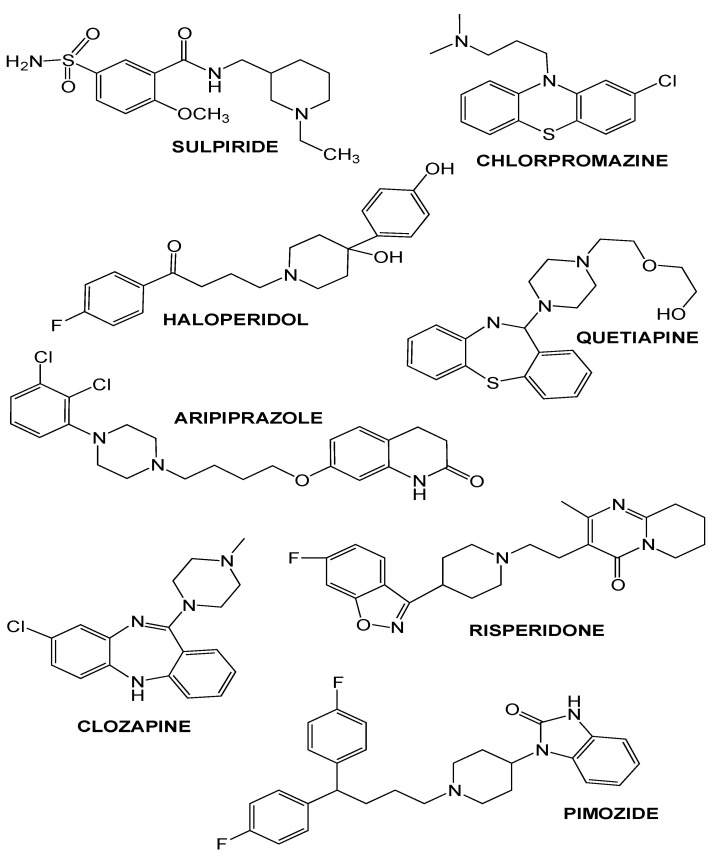
Representative chemical structures of both typical and atypical antipsychotics.

These findings consequently led to scheduling antipsychotics into two different classes. First, conventional (first-generation, typical) antipsychotics, which include among others chlorpromazine, haloperidol, pimozide, fluphenazine, and sulpiride (Figure 3, Table 2), targeting predominantly D2 dopamine receptors. Next, the second-generation neuroleptics, which include risperidone, olanzapine, quetiapine, ziprasidone, clozapine, and aripiprazole (Figure 3, Table 2) with more or less affinity to the serotonin 5-HT2 than the D2 receptor [55].

Interestingly, almost all atypical antipsychotics bind also to alpha-adrenergic receptors with the greatest affinity for clozapine, risperidone, iloperidone, and clozapine. Other atypical antipsychotics possess rather weak binding affinities toward muscarinic cholinergic receptors [57,58,59]. Furthermore, second-generation atypical antipsychotics have become more popular because they are believed to be effective in people with mental health problems who no longer respond well to primary treatment. Such new medications are suggested to reduce both negative and positive symptoms, such as hearing voices or seeing things, and troublesome undesirable adverse effects, such as sleepiness and EPS (e.g., tremors, stiff muscles, or involuntary facial movements) [60]. However, recent studies have indicated that the use of atypical antipsychotics is associated with a greater risk of developing metabolic syndrome—including diabetes, weight gain, and hyperlipidemia—which may be in the long-term perspective more problematic than EPS or tardive dyskinesia. These adverse effects are relevant for cardiovascular morbidity and mortality and are especially prominent early on in treatment and especially with clozapine and olanzapine. Nevertheless, looking at the side effects resulting from certain antipsychotics, it is known that there is much greater variability among each drug than if we were only analyzing class-to-class (first- vs. second-generation) differences.

## 5. Antipsychotics in the Treatment of DXM-Induced Psychoses

Management of DXM intoxication focuses mostly on symptom resolution. Sedation with medication is commonly required to control agitation, violent behavior, and psychosis due to DXM toxicity. Previously reported psychopharmacologic treatments of acute psychotic symptoms induced by DXM include low-dose antipsychotics (haloperidol, risperidone, and quetiapine) [33]. In addition, limited controlled clinical trials have shown that olanzapine has a better efficacy and safety profile than haloperidol and, according to some studies, seems to be more efficient in the management of negative symptoms [61,62,63,64]. More importantly, parenteral antipsychotics (e.g., haloperidol) should be avoided in patients with DXM overdose because they may exacerbate hyperthermia or anticholinergic symptoms.

Martinak and colleagues reported a case of a 40-year-old female chronically using DXM at very high doses (from 1080–1620 mg/day to 3000–4000 mg/day), who was effectively treated with olanzapine [14]. According to Martinak et al. [14], a combination of both olanzapine and a mood stabilizer (delayed-release divalproex sodium) was effective in the control of severe psychotic symptoms induced by DXM abuse. Noteworthy, in one of the studies, a man who abused DXM with auditory hallucinations was effectively treated with risperidone at a dose of 6 mg nightly [65]. Similar findings were demonstrated by Logan and co-workers [33]. It is important to consider that both risperidone and olanzapine have been shown to be well-tolerated in the treatment of psychotic disorders [66]. Of note, findings have also demonstrated that complete remission without neuroleptics after discontinuing the abuse of DXM is possible as well [13,45].

Given the above findings and the neurobiological basis of drug-induced psychoses (Figure 2), antagonism of dopamine type 2 (D2) and serotonin 2A (5HT2A) receptors and high binding affinity may be an advantage of atypical antipsychotics in the treatment of DXM-related psychosis [67,68,69] (Table 3).

In the case of haloperidol, which has a low affinity for the 5-HT2A receptor (Table 3), antipsychotic efficacy can be mainly explained by the D2 blockade and inhibition of the CYP2D6 enzyme, since the drug can modulate DXM levels and change the neuropsychiatric presentation [70]. Other reports also demonstrated the relationship between CYP2D6 activity, plasma drug levels, psychoactive drug effects, and the abuse liability and therapeutic utility of DXM [71].

**Table 3:** Receptor selectivity of antipsychotic drugs.

Receptor	Haloperidol	Risperidone	Olanzapine	Quetiapine
D2	0.1	0.5	4.0	30
5-HT2A	50	0.2	2.5	250

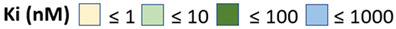
 Data are Ki values in nM derived from functional antagonist R-SAT™ assays. Abbreviations: Ki (nM)—binding affinity data; D2—dopamine 2 receptor; 5-HT2A—serotonin 2A receptor. According to [72] with modification.

However, to date, findings on this topic are limited and inconsistent. The interactions of DXM-antipsychotic drugs are probably underdiagnosed and under-reported [26]. Shin et al. [73] reported that haloperidol and its metabolites (i.e., dealkylated metabolites 4-(4-chlorophenyl)-4-hydroxypiperidine and *p*-fluorobenzoyl propionic acid) inhibit the human liver cytochrome P450 2D6 in vitro; thus, the DXM formulation rate was diminished. In another study, poor metabolism (PM) of DXM may cause greater accumulation and a higher risk occurrence of intolerance [74]. It has also been found that poor metabolizers using DXM reported greater sedation and dysphoria compared with extensive metabolizers [75].

The rational explanation for such a phenomenon is the diverse affinity of DXM and its main metabolite DEXO to both sigma-1 (Sig1-R) and NMDA receptors. DXM is a stronger Sig1-R agonist than DEXO, and DEXO is a stronger NMDA receptor antagonist than DXM. Thus, both of these compounds are capable of inducing psychoactive effects; however, DEXO may be a more reliable compound for the psychotic and dissociative states [33,37,42,76]. Indeed, since poor metabolizers produce less DEXO and maintain elevated levels of DXM, they experience more pronounced side effects, such as nausea, vomiting, dysphoria, sedation, and, subsequently, have less potency for abuse. The frequency of the PM phenotype of DXM in Caucasian populations varies between 5 and 10% [74].

In contrast, since extensive metabolizers produce more DEXO, which has a higher affinity for the NMDA receptor than DXM, they experience psychoactive and euphoric effects and present with a higher risk for abuse [75]. This action results in neurobehavioral effects similar to ketamine and PCP, including hallucinations, “out of body” sensations, and dissociation. Approximately 85 percent of the U.S. population has high CYP2D6 activity and would be expected to rapidly develop a high DEXO level after overdose. The aforementioned findings provide preliminary evidence that DEXO contributes to DXM-abuse liability, and therefore, poor metabolizers may be less likely to abuse DXM [71,75].

## 6. Conclusions

Taken together, a long history of DXM presence on the market is a good example of how OTC drugs, considered relatively safe, may induce serious complications. Noteworthy, DXM is safe only when it is used properly, according to the officially approved summary of the medicinal product (SmPC). However, its recreational or repeated use, especially by teenagers and young adults, should be considered as the main risk of psychotic symptoms (i.e., mania, psychosis, or hallucinations). Taking this into consideration, all patients should receive complete information from general practitioners about the potentially serious adverse effects of DXM. In addition, a nationwide ban on DXM sales for teens to minors would lead to a greater decrease in the abuse rate of these medications while maintaining access for the millions of legitimate consumers of these products each year.

Atypical antipsychotic agents (i.e., risperidone, olanzapine) were found to be beneficial in the symptomatic treatment of DXM-related psychosis, mostly due to their better efficacy and safety profile than typical haloperidol in the short-term course. However, the literature on DXM-related psychosis generally exhibited several limitations. It is worth emphasizing that, due to the numerous limitations, the presented article is a narrative review. The first limitation is that there are no high-credibility randomized clinical trials as well as meta-analyses evaluating and comparing the efficacy of antipsychotic drugs in the treatment of DXM-related psychosis; only limited case reports and case series studies have been published on this topic so far. Therefore, given the lack of controls and concurrent use of other antipsychotics, it is not yet sufficient to propose general information about their efficacy for the clinical improvement of DXM-related psychosis. In addition, the currently available data are not yet sufficient to propose a general hypothesis. Thus, the presented article is rather speculative and does not reflect the full scope of the health benefits resulting from the use of antipsychotics to treat DXM-induced psychosis. Further clinical studies with high-quality data and large sample sizes are required to validate these findings. Second, the situation is complicated by the fact that many authors failed to report all ingredients and strengths that were involved in their reported cases, and mixed poisonings are much more difficult to treat. Therefore, the influence of coingestants should be adequately evaluated to estimate the real potency of atypical antipsychotics in DXM-induced psychosis management.

## Figures and Tables

**Table 1:** Dextromethorphan intoxicating levels (adapted with permission from [36]. 2008, Krajowe Centrum Przeciwdziałania Uzależnienom).

DXM-Induced Intoxication Level	Dose [mg/kg b.w]	Effects
I plateau	1.5–2.5	mild intoxication such as after alcohol use slight stimulation such as after caffeine use improvement in well-being, euphoria with an impression of being united with the world the disturbed feeling of gravity improvement of the perception of sound stimuli
II plateau	2.5–7.5	stronger symptoms as in the I plateau visual hallucinations (with eyes closed) locomotor disturbance speech disorders locomotor disturbance (“robotic walk”, “zombie walk”) impairment of perceptual functioning, comparable to the combined use of alcohol and cannabis
III plateau	7.5–15.0	the feeling of strong anesthesia disturbances of consciousness disturbance of the functions of the senses, mainly eyesight (e.g., desynchronization of the perception of visual impressions) a mild sensation of separation from the body (dissociation) similar to low-dose ketamine use
IV plateau	>15	a strong sensation of separation from the body (dissociation) depersonalization, sensation of death associated with anesthesia of the body hallucinations experiences partially covered by amnesia, some of them recalled after a few days symptoms resemble those of ketamine and PCP violent behaviors, elevated temperatures, possible death from cardiac or respiratory arrest

**Table 2:** Representative typical and atypical antipsychotic drugs with their potential mechanism of action.

Antipsychotics		Mechanism of Action (Target Receptors) *
First-Generation Class (Typical, Conventional)	Haloperidol	Primarily via D2 antagonism
	Droperidol	
	Fluphenazine	
	Chlorpromazine	
	Thioridazine	
	Thiothixene	
	Loxapine	
	Molindone	
	Trifluoperazine	
	Perphenazine	
Second-Generation Class (Atypical)	Olanzapine	Primarily via D2 (weak), D4, and 5HT2A antagonism; also, through α-adrenergic, muscarinic, and histamine receptors
	Risperidone	
	Ziprasidone	
	Clozapine	
	Quetiapine	
	Aripiprazole	
	Asenapine	
	Zotepine	
	Sulpiride	
	Paliperidone	
	Iloperidone	
	Lurasidone	
	Levosulpiride	
	Amisulpride	
	Perazine	
	Sertindole	

* The binding affinities data were adapted from [56].

## Data Availability

Not applicable.
